# Exposure to podophyllotoxin inhibits oocyte meiosis by disturbing meiotic spindle formation

**DOI:** 10.1038/s41598-018-28544-1

**Published:** 2018-07-05

**Authors:** Lin-Lin Hu, Xin Zhou, Hao-Lin Zhang, Lan-Lan Wu, Lian-Sheng Tang, Ling-Li Chen, Jin-Liang Duan

**Affiliations:** 1 Center for Reproduction, 181st Hospital of Chinese People’s Liberation Army, Guilin, 541001 China; 2 College of Animal Science and Technology, Nanjing Agricultural University, Nanjing, 210095 China; 3 Shandong Institute of Pharmaceutical Industry, Shandong Provincial Key Laboratory of Chemical Drugs, Jinan, 250101 China

## Abstract

Podophyllotoxin is used as medical cream which is widely applied to genital warts and molluscum contagiosum. Although previous study showed that podophyllotoxin had minimal toxicity, it was forbidden to use during pregnancy since it might be toxic to the embryos. In present study we used mouse as the model and tried to examine whether podophyllotoxin exposure was toxic to oocyte maturation, which further affected embryo development. Our results showed that podophyllotoxin exposure inhibited mouse oocyte maturation, showing with the failure of polar body extrusion, and the inhibitory effects of podophyllotoxin on oocytes was dose-depended. Further studies showed that the meiotic spindle formation was disturbed, the chromosomes were misaligned and the fluorescence signal of microtubule was decreased, indicating that podophyllotoxin may affect microtubule dynamics for spindle organization. Moreover, the oocytes which reached metaphase II under podophyllotoxin exposure also showed aberrant spindle morphology and chromosome misalignment, and the embryos generated from these oocytes showed low developmental competence. We also found that the localization of p44/42 MAPK and gamma-tubulin was disrupted, which further confirmed the effects of podophyllotoxin on meiotic spindle formation. In all, our results indicated that podophyllotoxin exposure could affect mouse oocyte maturation by disturbing microtubule dynamics and meiotic spindle formation.

## Introduction

Mammalian oocyte maturation is an important cellular process which ensures the oocyte quality for the fertilization and early embryo development^1^. The meiosis resumption begins with germinal vesicle break down (GVBD), and then α-tubulin and β-tubulin form microtubules. The microtubules assemble to form the meiotic spindle under the regulation of centrosome proteins such as γ-tubulin, which leads the chromosome congression under the monitor of a mechanism called spindle assembly checkpoint (SAC)^2^. Then actin filaments drive the meiotic spindle to the oocyte cortex. Finally the oocytes extrude their first polar bodies waiting for fertilization. Cytoskeleton assembly is critical for oocyte maturation. The disorder of microtubule dynamics will cause the spindle formation defects, which further induces the chromosome misalignment and aneuploidy. While the disassembly of actin filaments will cause the failure of oocyte asymmetric division, which further disturbs embryo development. During oocyte maturation, several molecules are shown to be involved in cytoskeleton dynamics and cell cycle control, for example, MEK and MAPK are shown to be involved into spindle formation in mouse oocytes^3^; Bub and Mad family members like BubR1, Bub3 and Mad2 together with chromosomes passenger complex (CPC) monitor SAC in oocytes^4^; and small GTPases RhoA, Cdc42, Rac1 and actin nucleator ARP2/3 complex regulate actin assembly to ensure the asymmetric division in mouse oocytes^5,6^.

Podophyllotoxin is a lignan extracted from podophyllum genera like podophyllum, Dysosma, Diphylleia and Juniperus. Podophyllotoxin is used as a medical cream which is widely applied to genital warts and molluscum contagiosum^7^. Podophyllotoxin is also adopted as a novel potential natural anticancer agent^8^. Till now podophyllotoxin is shown to have minimal toxicity. However, the poisoning cases are reported like manifested nausea and vomiting; moreover, the adverse effects like skin reactions including pain and burning are also shown in the previous studies^9^. Moreover, podophyllotoxin is shown to inhibit an early entry step of human cytomegalovirus^10^. Curcumin is reported to have protective effects on podophyllotoxin toxicity^11^. And the studies for the working mechanisms of podophyllotoxin effects from the cellular level show that podophyllotoxin mainly inhibits the microtubule dynamics^12^. Podophyllotoxin has similar effects with vinblastine and nocodazole on mitotic spindles, since podophyllotoxin treatment causes mitotic arrest accompanied by microtubule depolymerization^13^.

In present study we wondered whether podophyllotoxin could affect mammalian oocyte maturation, which further caused the embryo defects, since the aneuploidies in embryos are generally due to oocyte maturation disorders^14^. And we investigated the effects of podophyllotoxin on spindle formation and chromosome alignment during mouse oocyte maturation. Our results indicated the toxic effects of podophyllotoxin on meiotic spindle organization, which provided one possible reason for the forbidden use of podophyllotoxin during pregnancy from subcellular level.

## Results

### Podophyllotoxin exposure affects mouse oocyte maturation in a dose-manner

**Figure 1:**
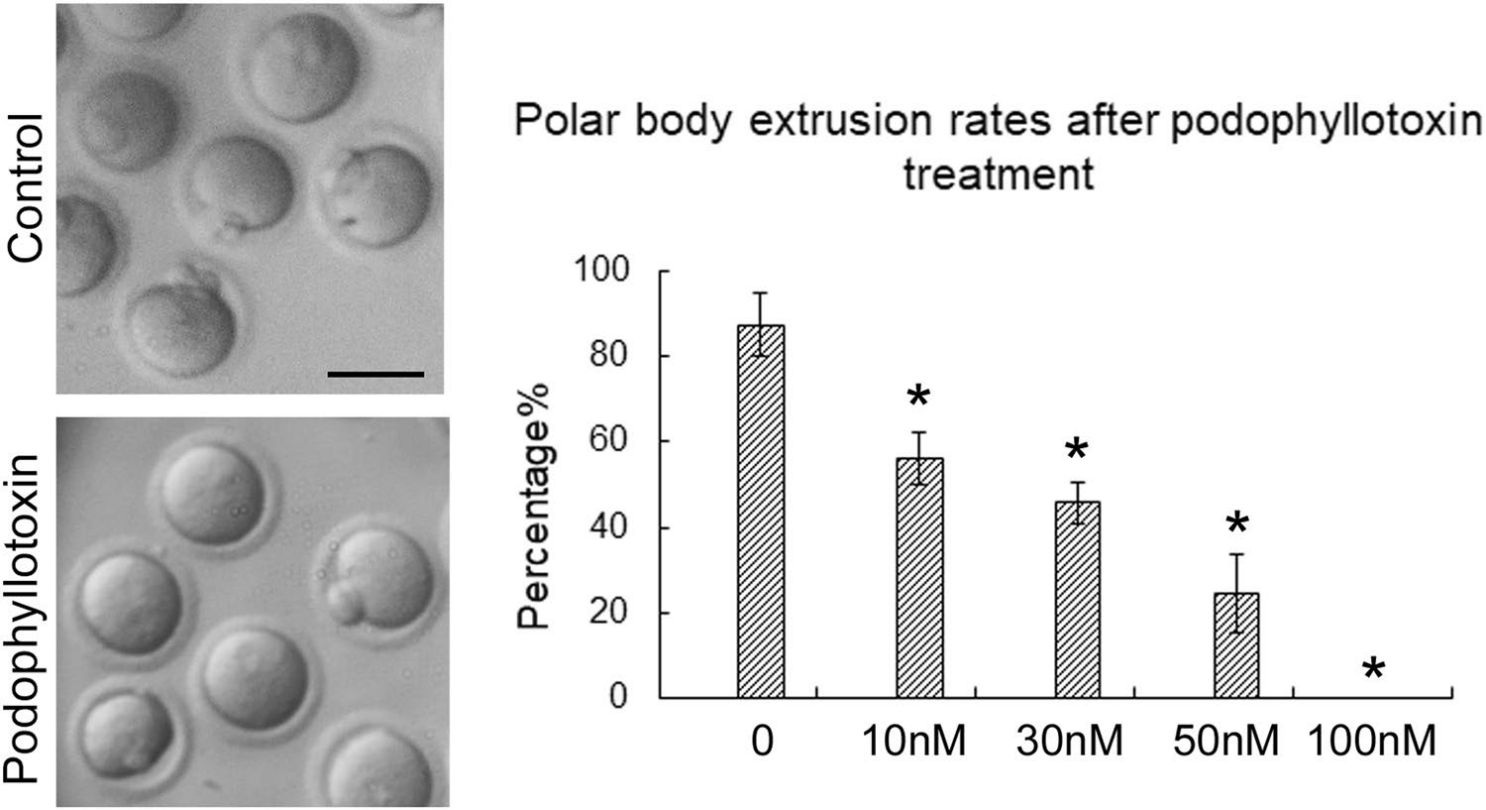
Podophyllotoxin exposure affected mouse oocyte maturation. The oocytes were cultured with 10 nM, 30 nM, 50 nM and 100 nM podophyllotoxin for 12 h. Most oocytes extruded the first polar body in the control group, however, a big proportion of oocytes failed to extrude the polar body in the podophyllotoxin treatment group. The polar body extrusion rate was significantly decreased with podophyllotoxin treatment in a dose dependent manner. *The statistical test was performed (p < 0.05).

We first examined the exposure of podophyllotoxin on mouse oocytes. And we found that podophyllotoxin significantly inhibited the maturation of mouse oocytes, most oocytes could not extrude their polar bodies (Fig. 1, 30 nM). We then did the dose test, and the results showed that 10 nM podophyllotoxin exposure already caused the significantly decrease for the rate of polar body extrusion during mouse oocytes maturation (87.3 ± 7.3%, n = 153 versus 56.1 ± 6.3%, n = 132; p < 0.05). Similar results were found in 30 nM (45.7 ± 4.8%, n = 101; p < 0.05), 50 nM (24.6 ± 9.3%, n = 121; p < 0.05) and 100 nM (0, n = 144; p < 0.05) podophyllotoxin treatment group. Our results showed that the effects of podophyllotoxin on mouse oocyte maturation was dose-depended (Fig. 1). 30 nM podophyllotoxin treatment was adopted in our following study.

### Podophyllotoxin exposure affects spindle formation and chromosome alignment in oocyte meiosis I

**Figure 2:**
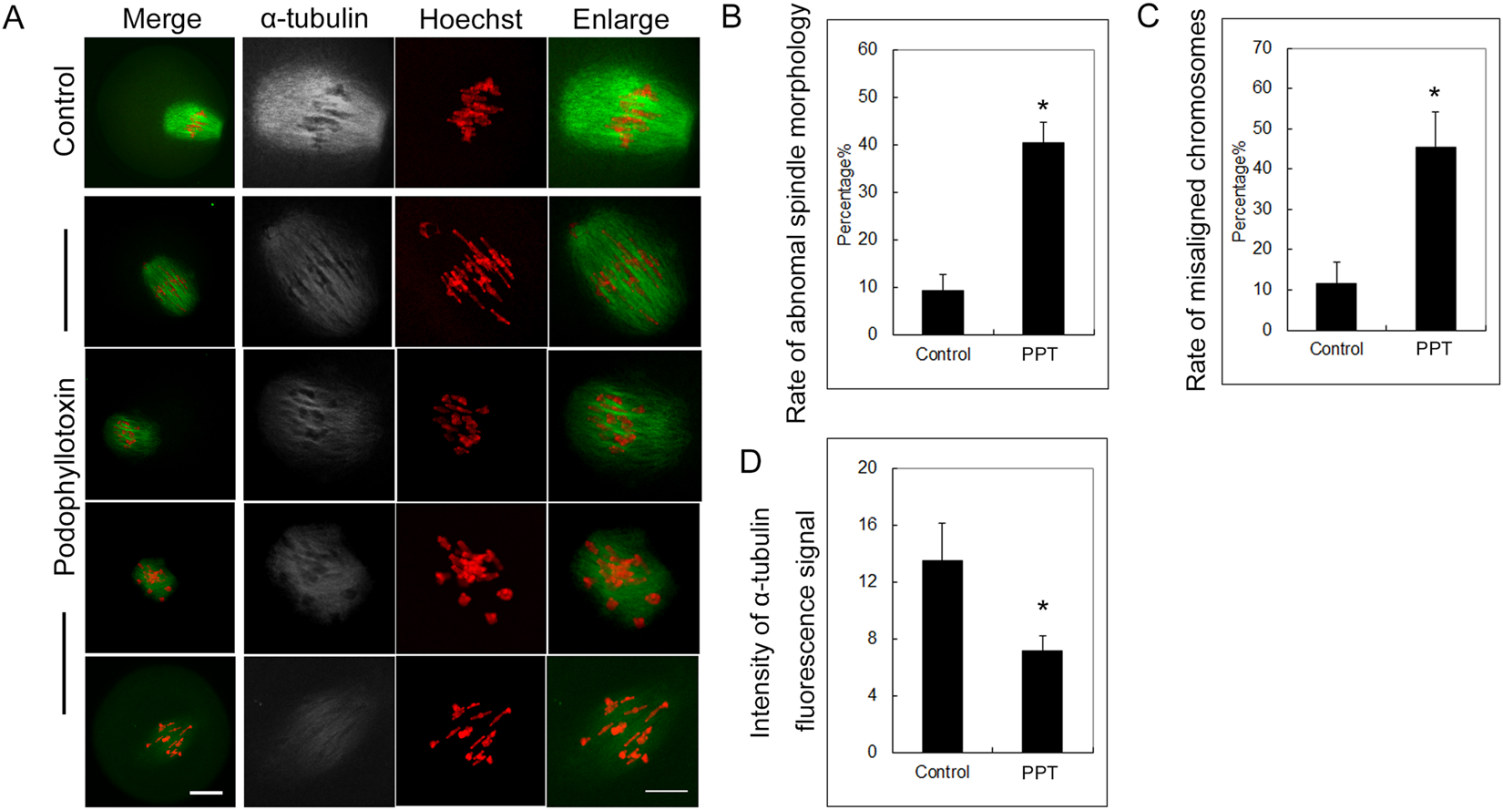
Podophyllotoxin exposure affected spindle formation and chromosome alignment in the meiosis I of oocytes. (**A**) Fluorescence staining for the spindle and chromosomes after podophyllotoxin exposure. In the control group, the spindle showed well barrel shape morphology, and the chromosomes aligned well. However, after podophyllotoxin exposure the spindle morphology was abnormal, the chromosomes were mis-aligned. Moreover, the fluorescence signals of microtubule were much weaker compared with the control group. Green, alpha-tubulin; red, chromosomes. Bar = 20 μm. (**B**) Rates of abnormal spindle morphology in the podophyllotoxin exposure oocyte group. *The statistical test was performed (p < 0.05). (**C**) Intensity of alpha-tubulin fluorescence signals in the podophyllotoxin exposure oocyte group. *The statistical test was performed (p < 0.05). (**D**) Rates of misaligned chromosomes in the podophyllotoxin exposure oocyte group. *The statistical test was performed (p < 0.05).

Our results above indicated that podophyllotoxin was toxic to mouse oocyte maturation. We then tried to explore the potential reasons for the low developmental competence of oocytes after 30 nM podophyllotoxin treatment. We first examined the formation of meiotic spindle in meiosis I. After culturing the oocytes for 8.5 h, the time points that most mouse oocytes reach metaphase I, the meiotic spindle formed in the control oocytes, showing with typical barrel-shape structure and well-aligned chromosomes. However, we found that most oocytes showed aberrant spindle morphology after 30 nM podophyllotoxin treatment, and the chromosomes were misaligned, some chromosomes even moved to the poles of spindle. Moreover, the fluorescence signals of alpha-tubulin were much weaker compared with the control group (Fig. 2A). We also analyzed the abnormal rates of spindle morphology and chromosome alignment, and the statistic results showed that the abnormal rates of spindle morphology in 30 nM podophyllotoxin treatment group were all significantly higher that the control group (For spindle morphology, 40.4 ± 4.3%, n = 101 versus 9.2 ± 3.5%, n = 87; p < 0.05, Fig. 2B). And for chromosome alignment, similar result was also found (45.3 ± 8.8%, n = 101 versus 11.5 ± 5.4%, n = 87, p < 0.05. Fig. 2C). To further confirm this, we quantified the fluorescence signal intensity of alpha-tubulin, and we found that the alpha-tubulin signals were significantly lower compared with the control group (7.1 ± 1.1 versus 13.5 ± 2.6, n = 30; p < 0.05) (Fig. 2D). These results indicated that podophyllotoxin treatment disrupted spindle formation and chromosome alignment in meiosis I.

### Podophyllotoxin exposure affects spindle formation and chromosome alignment in oocyte meiosis II

**Figure 3:**
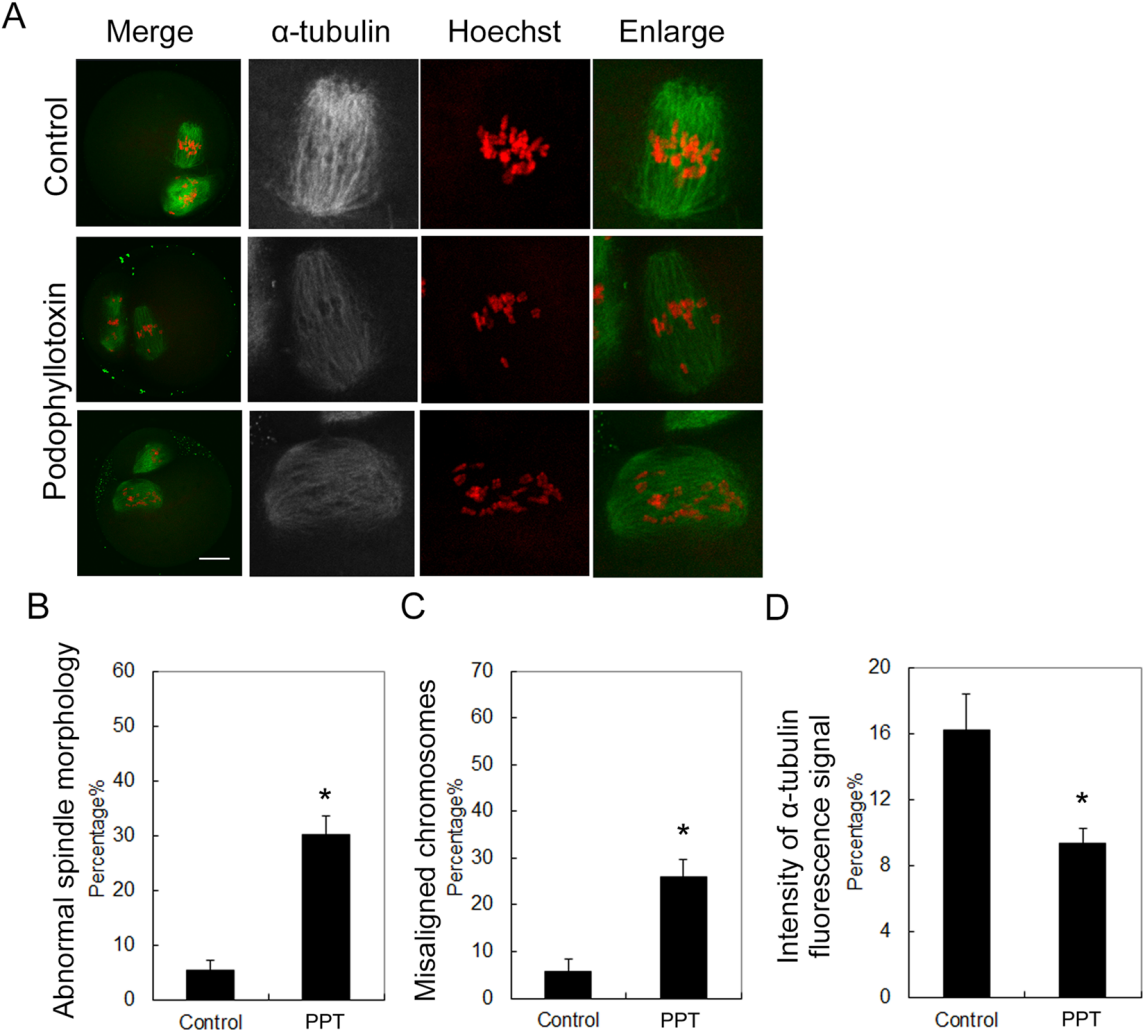
Podophyllotoxin exposure affected spindle formation and chromosome alignment in meiosis II of oocytes. (**A**) Fluorescence staining for the spindle and chromosomes after podophyllotoxin exposure. In the control group, the oocytes extruded the first polar body, the spindle showed well barrel shape morphology, and the chromosomes aligned well. However, after podophyllotoxin exposure the spindle morphology was disrupted, the chromosomes were mis-aligned. Similar with meiosis I, the fluorescence signals of microtubule were much weaker compared with the control group. Green, alpha-tubulin; red, chromosomes. Bar = 20 μm. (**B**) Rates of abnormal spindle morphology in the podophyllotoxin exposure oocyte group. *The statistical test was performed (p < 0.05). (**C**) Intensity of alpha-tubulin fluorescence signals in the podophyllotoxin exposure oocyte group. *The statistical test was performed (p < 0.05). (**D**) Rates of misaligned chromosomes in the podophyllotoxin exposure oocyte group. *The statistical test was performed (p < 0.05).

Our results indicated that more than a half of oocytes failed to extrude the polar body after podophyllotoxin treatment. This suggested that almost a half oocytes still could extrude the polar body under podophyllotoxin exposure. We tried to examine whether these oocytes with polar body extrusion were affected by the podophyllotoxin treatment. We cultured the oocytes for 12 h and most oocytes were arrested at metaphase II (MII) in the control group, showing with polar bodies with well-formed spindles and chromosome alignment. However, similar with our findings in meiosis I, although these oocytes still could extrude the polar bodies under 30 nM podophyllotoxin treatment, the spindle morphology was also aberrant and the chromosomes were severely misaligned in these MII oocytes (Fig. 3A). The statistical analysis data also confirmed this (for spindle morphology, 30.2 ± 3.3%, n = 47 versus 5.3 ± 1.8%, n = 65; p < 0.05, Fig. 3B; for chromosome alignment, 25.9 ± 3.9%, n = 47 versus 5.8 ± 2.5%, n = 65, Fig. 3C). Moreover, the fluorescence intensity of microtubules was also decreased after 30 nM podophyllotoxin treatment (9.3 ± 0.9 versus 16.2 ± 2.2, n = 30; p < 0.05) (Fig. 3D). These results indicated that podophyllotoxin treatment could also disrupt spindle formation and chromosome alignment in meiosis II.

### Podophyllotoxin exposure disrupts MAPK and gamma-tubulin localization in mouse oocytes

**Figure 4:**
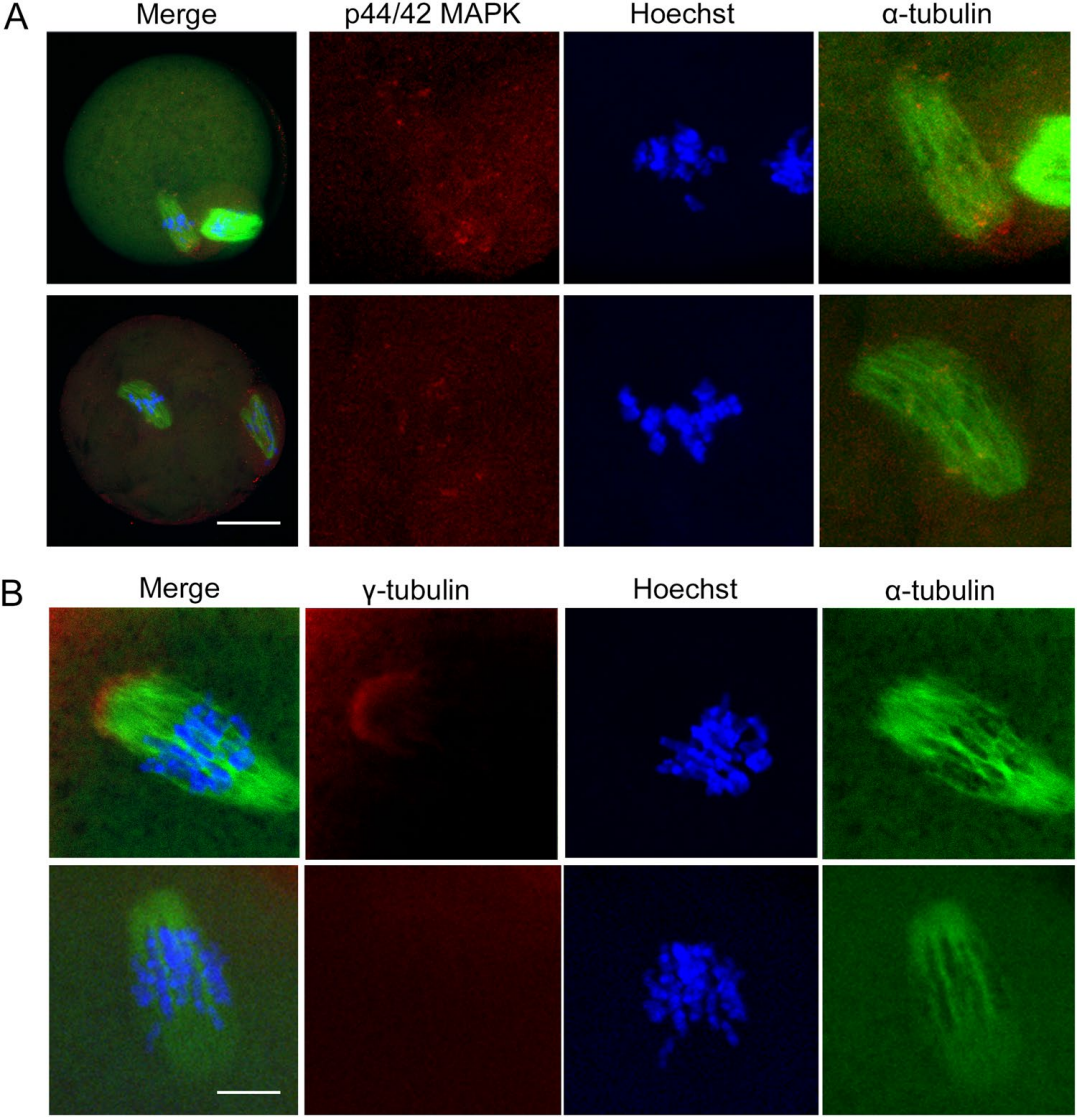
Podophyllotoxin exposure affected the localization pattern of p44/42 MAPK and gamma-tubulin in mouse oocytes. (**A**) In the control oocytes, p44/42 MAPK localized at the poles of spindle, while in the podophyllotoxin treatment oocytes, the signals of p44/42 MAPK scattered around the spindle. Green, alpha-tubulin; red, p44/42 MAPK; blue, chromosomes. Bar = 20 μm. (**B**) In the control oocytes, gamma-tubulin localized at the poles of spindle, while in the podophyllotoxin treatment oocytes, the signals of gamma-tubulin dispersed from the spindle. Green, alpha-tubulin; red, gamma-tubulin; blue, chromosomes. Bar = 5 μm.

To further confirm that disruptive effects of podophyllotoxin on meiotic spindle formation we then explored the localization pattern of p44/42 MAPK and gamma-tubulin after podophyllotoxin treatment. As shown in Fig. 4A, in the control oocyte, p44/42 MAPK localized at the poles of spindle, while in the 30 nM podophyllotoxin treatment oocyte the fluorescence signals of p44/42 MAPK scattered around the twisted meiotic spindle. We also examined the localization of gamma-tubulin, which was shown to be accumulated at the poles of spindle, however, in the 30 nM podophyllotoxin treatment oocytes there is barely gamma-tubulin signals (Fig. 4B). This result suggested that podophyllotoxin treatment disrupted the localization of spindle regulators.

### The oocytes under the podophyllotoxin exposure fail to develop to early embryos

**Figure 5:**
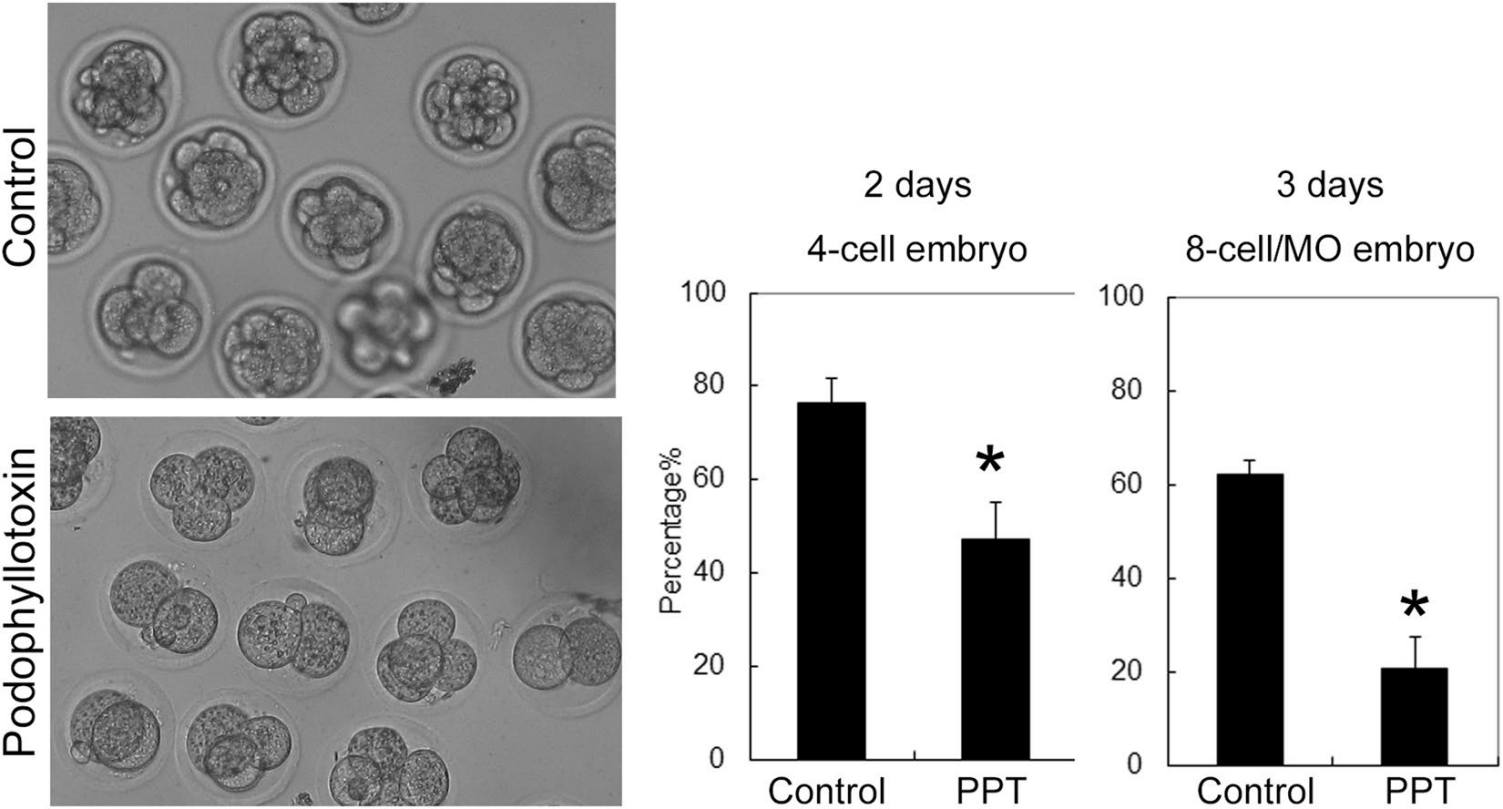
Podophyllotoxin exposure affected embryo development competence. The oocytes were cultured 30 nM podophyllotoxin for 12 h. The oocytes which extruded the first polar body were collected. And the embryos generated from the parthenogenetic activation oocytes with podophyllotoxin treatment group showed lower developmental rates after 2 days and 3 days culture. *The statistical test was performed (p < 0.05).

Our results above indicated that 30 nM podophyllotoxin treatment caused more than a half of oocytes failed to extrude the first polar bodies, which indicated that almost a half oocytes still could undergo maturation. We wondered whether these oocytes could develop to the blastocyst embryos. We performed parthenogenetic activation for these podophyllotoxin exposure oocytes and we found that after two days culture, the rate of 4-cell embryos from 30 nM podophyllotoxin treated oocytes was significantly lower than the control group (47 ± 8.2%, n = 82 versus 76.2 ± 5.5%, n = 96; p < 0.05); while after 3 days culture, the rate of 8-cell/morula embryos from 30 nM podophyllotoxin treated oocytes was also significantly lower than the control group (20.7 ± 6.8%, n = 73 versus 62.2 ± 2.8%, n = 92; p < 0.05) (Fig. 5). The results indicated that the embryos generated from podophyllotoxin treated oocytes had lower developmental competence.

## Discussion

In present study, we examined the effects of podophyllotoxin exposure on the mouse oocyte maturation. We explored the microtubule assembly, meiotic spindle organization and chromosome alignment in mouse oocytes after treatment with podophyllotoxin. Our results indicated that podophyllotoxin had toxic effects on meiotic spindle formation, which further affected mouse oocyte maturation.

We first found that podophyllotoxin inhibited mouse oocyte meiosis, showing with the failure of polar body extrusion, the typical feature for the oocyte maturation. Podophyllotoxin was shown to inhibit mitosis, for example, podophyllotoxin treatment was shown to induce mitotic arrest and pro-apoptoic ER stress in lung cancer^15^. Moreover, podophyllotoxin could promoting cell death via cell cycle arrest, ER stress and autophagy^16^. Our results was consistent with mitosis, indicating the toxic effects of podophyllotoxin on female meiosis. Similar phenotype was also found in other toxins like mycotoxin members. For example, zearalenone and HT-2 toxin could inhibit the mouse, pig and zebrafish oocyte maturation and early embryo development^17–19^.

To find out the causes for the oocyte maturation defects after podophyllotoxin treatment. We first examined the spindle formation, the critical cellular step for the chromosome segregation during meiosis I. We found that in the podophyllotoxin treatment group the oocytes showed abnormal spindle morphology, indicating that podophyllotoxin might affect spindle formation in mouse oocytes. Moreover, we also found that podophyllotoxin treatment disrupted chromosome alignment. These two phenotypes might be all due to the effects of podophyllotoxin on microtubule dynamics in oocytes, since the microtubule assembly is critical for meiotic spindle formation, which further leads to the chromosome congression. In mitosis, podophyllotoxin was also shown to mainly affect microtubule dynamics for the mitotic arrest. Podophyllotoxins was even used in the sea urchin embryo as microtubule destabilizing agents^20^. Podophyllotoxins was also shown to inhibit microtubule assembly on glima cell proliferation and human cancer cells^21,22^. Similar with podophyllotoxins, lots of toxins could affect oocyte maturation through their effects on microtubule assembly and spindle formation. The mycotoxin family member ZEN, DON and T-2 toxin could inhibit mouse oocytes by disrupting spindle formation^23–25^. The disruptive effects of ZEN and DON were also reported on microtubule dynamics in other species like pig oocytes^26,27^. These previous studies together with our work indicated that the effects of toxins on spindle organization might be one important way to affect both mitosis and meiosis. Moreover, our results also showed that spindle morphology and chromosome alignment were all abnormal in oocytes which under the podophyllotoxins exposure. And the embryos generated from these oocytes failed to develop to 8-cell or morula stage. This indicated the widely disruptive effects of podophyllotoxins on both meiosis I and meiosis II of oocytes, which further affected early embryo development.

To further confirm our hypothesis, we also examined the localization pattern of spindle organization regulators MAPK and gamma-tubulin. These two molecules were reported to regulate meiotic spindle formation in both male and female meiosis^1^. Our results indicated that these two molecules all failed to accumulate at the poles of spindle, and this might be due to the disruption of spindle morphology after podophyllotoxin treatment, which also confirmed our hypothesis that podophyllotoxin caused the defects of spindle organization. A question is also raised that whether podophyllotoxin regulates MAPK or gamma-tubulin for spindle formation. Recently a study indicated that podophyllotoxin induced CREB phosphorylation and CRE-driven gene expression via PKA but not MAPKs^28^. While our results showed no difference for the fluorescence intensity of MAPK or gamma-tubulin after podophyllotoxin exposure (statistics data not shown), which was consistent with this study. However, how podophyllotoxin affects microtubule assembly still needs further mechanism study.

In summary, our results indicated that podophyllotoxin might affect microtubule dynamics, which caused the spindle formation and chromosome alignment for mouse oocyte maturation, and this might be the causes for the low developmental competence of embryos exposed with podophyllotoxin.

## Materials and Methods

### Antibodies and chemicals

Podophyllotoxin was bought from J&K Chemical Ltd. (Shanghai, China. Cat. 131142). Mouse monoclonal anti-α-tubulin-FITC antibody, Hoechst 33342 were purchased from Sigma (St Louis, MO, USA). Anti-gamma-tubulin antibody was from the Abcam (Cambridge, UK). Rabbit anti-p44/42 MAPK antibody was purchased from Cell Signaling Technology (Boston, MA).

### Ethic statement and oocyte culture

All animal experiments were conducted in accordance with the guidelines of the Animal Research Committee of Nanjing Agricultural University, China (XYXK-Su-2017-0007). 3 months ICR female mice (Beijing Weitonglihua Experimental Animal Center were used). Mice were housed in a temperature-controlled room with regular daily dark-light cycles, fed a regular diet (mouse feeds from Qinglongshan Experimental Animal Corporation, Nanjing), and maintained under the care of the Experimental Animal Center, Nanjing Agricultural University, China. The mice were sacrificed by cervical dislocation. And this study was approved by the Committee of Animal Research, Nanjing Agricultural University. Germinal vesicle stage oocytes were harvested from mouse ovaries and were cultured in M16 culture medium (Sigma) with 37 degree and 5% CO_2_.

### Parthenogenetic activation

After 12 h, the oocytes with polar bodies were selected for the incubation in activating medium for 6 min at room temperature. The activating medium was M16 which containing 7% ethanol. After activating, the oocytes were washed with KSOM medium (Merck), and then they were incubated in KSOM at 37 °C in a humidified atmosphere with 5% CO_2_ for culture to the 8-cell/morula stage.

### Podophyllotoxin treatment

Podophyllotoxin (100 mg, J&K Chemical Ltd. Cat. 131142) was dissolved in dimethyl sulfoxide (DMSO) to a final concentration of 50 mM for storage. And the GV oocytes were cultured in 10–100 nM podophyllotoxin in M16 medium (Sigma) at 37 °C in a humidified atmosphere with 5% CO_2_. The control oocytes were cultured in fresh M16 medium with the same concentration of DMSO. After different culture time, the mouse oocytes were collected for the fluorescence staining.

### Confocal microscopy analysis

The sample oocytes were collected with different culture time, and then were fixed with 4% paraformaldehyde in phosphate-buffered saline (PBS) at room temperature for 30 mins, and then the oocytes were moved to the membrane permeabilization solution (0.5% Triton X-100) for 30 mins. After the 1 hour incubation in PBS with 1% BSA (blocking buffer) at room temperature, the oocytes were incubated with the first antibody (α-tubulin-FITC, p44/42 MAPK or gamma-tubulin) for 4 h. Then the oocytes were washed with phosphate-buffered saline (PBS), and then were moved to the secondary antibodies for 1 h (α-tubulin-FITC excluded). Oocytes were stained with Hoechst 33342 for 20 mins. And the oocytes were mounted on glass slides and were scanned with confocal microscope (Zeiss LSM 700 META; Jena, Germany). We repeated at least three times and at least 30 oocytes were examined.

### Fluorescence intensity analysis

For the analysis of the fluorescence intensity of tubulin fluorescence signals. All the control and treated oocytes were mounted on the one same glass slide. After the acquirement of pictures from confocal microscope. Image J software (NIH) was used to measure the region of interest (ROI), one ROI was measured in one oocyte and the average fluorescence intensity for every unit area within the ROI was calculated. ROIs were taken for the measurement of spindle area. 10 oocytes were analyzed in each repeat. The average values of all measurements were used to calculate the final average intensities between control and treated oocytes.

### Statistical analysis

For each experiment group, at least three biological repeats were done with the data expressed as means ± SEMs. Statistical analysis were performed by one-way ANOVA with a means separation test and independent-sample t-tests. A p-value of <0.05 was considered significant.

### Availability of data

All data generated or analyzed in this study are included in this published article.
